# The evolution of mendelian randomization for investigating drug effects

**DOI:** 10.1371/journal.pmed.1003898

**Published:** 2022-02-03

**Authors:** Dipender Gill, Stephen Burgess

**Affiliations:** 1 Department of Epidemiology and Biostatistics, School of Public Health, Imperial College London, London, United Kingdom; 2 MRC Biostatistics Unit, School of Clinical Medicine, University of Cambridge, Cambridge, United Kingdom; 3 Department of Public Health and Primary Care, School of Clinical Medicine, University of Cambridge, Cambridge, United Kingdom

## Abstract

Dipender Gill and Stephen Burgess discuss the accompanying study by James Yarmolinsky and colleagues investigating the associations between genetically-proxied inhibition of antihypertensive drug targets and risk of common cancer subtypes using Mendelian randomization.

## Leveraging genetic variants that proxy drug effects

Proteins represent the majority of drug targets; therefore, genetic variants affecting the function or expression of genes encoding these proteins can be used as proxies for investigating the effect of pharmacologically perturbing the corresponding protein drug target [1]. Random allocation of genetic variants through meiosis and conception means that the genotype an individual inherits is not typically affected by environmental confounding factors or reverse causation, analogous to treatment allocation in a randomized controlled trial. Provided that the genetic proxy can only influence an outcome through its effect on the protein drug target and not some pleiotropic pathway, a genetic association with the outcome can serve as evidence for a potential effect of drug target perturbation on that outcome. This paradigm spawned the field of “drug target mendelian randomization,” which has now been used to prioritize the design of clinical trials for more than a decade [2]. In the accompanying study in *PLOS Medicine* by Yarmolinsky and colleagues [3], genetic variants were identified to proxy the effect of different antihypertensive drug classes and were leveraged in drug target mendelian randomization analyses to explore effects on risk of common cancer subtypes.

## Angiotensin converting enzyme inhibitors and risk of colorectal cancer

Using genetic variants related to blood pressure at the *ACE* gene, the authors found genetic evidence supporting an effect of angiotensin converting enzyme (ACE) inhibition on increased risk of colorectal cancer in UK Biobank participants of European genetic ancestry. Through colocalization analyses, the authors went on to show that a shared variant within the *ACE* locus was likely to be related to both circulating ACE protein levels and colorectal cancer risk. This is a critical experiment for corroborating the findings of mendelian randomization analyses performed to investigate the effect of drug target perturbation because it provides evidence to support that any identified association is not attributable to genetic confounding through a variant in linkage disequilibrium [4]. While the association between genetically proxied ACE inhibition and colorectal cancer risk replicated in the independent FinnGen consortium that is also made up of European genetic ancestry individuals, it was not observed when studying a Japanese population.

## Distinctions between genetic effects and pharmacological effects

While the observed association between genetically proxied ACE inhibition and increased colorectal cancer risk warrants further investigation [3], there are a number of important reasons why it should not currently affect clinical practice. Firstly, genetically proxied ACE inhibition (as based on variants at the *ACE* gene that associated with lower circulating ACE protein levels and lower blood pressure) was shown to be associated with increased *ACE* gene expression in the colon [3]. Indeed, it is common for the variants that predict expression of a given gene to vary between different tissues, sometimes with opposite directions of effect. This raises the possibility that ACE inhibition locally in the colon might actually protect against colon cancer, rather than increase its risk. Furthermore, it is not clear whether the pharmacological effect of ACE inhibitor drugs in clinical practice extend to the colon, an issue that is critical to resolving their potential effects on colorectal cancer risk. Secondly, genetic variants proxying drug effects represent the cumulative lifelong impact of a small degree of drug target perturbation, which contrasts a pharmacological intervention in later life that typically has a greater magnitude of effect for a shorter period of time [1]. It is for exactly this reason that mendelian randomization estimates of genetically proxied drug effects are typically greater in magnitude than those observed in clinical practice [5]. Finally, despite the detailed genetic investigations performed by the authors, there remains the possibility that the identified associations may be attributable to pleiotropic effects of the genetic variants that are unrelated to the pharmacological ACE inhibition achieved through medications used in clinical practice.

## Future perspectives

Taken together, these findings generated by Yarmolinsky and colleagues offer a number of potential insights and areas for future study. First, they raise the possibility that ACE inhibition may increase the risk of colorectal cancer, in turn highlighting the need for pharmacovigilance toward this association. Second, they offer possible mechanistic insight into the development of colorectal cancer, which may, in turn, reveal therapeutic opportunities. Third, the discrepancy in findings between individuals of European and East Asian genetic ancestry may suggest effects that are confined to specific ethnic groups, thus having implications for prescribing strategies.

The work also demonstrates some of the progress that has been made over the last decade in genetic analyses investigating drug effects. The complementary application of drug target mendelian randomization and colocalization analyses by Yarmolinsky and colleagues was able to strengthen the genetic evidence for causality through investigating whether it is the same genetic variant that underlies the observed associations with the exposure and the outcome. Other methodological developments have further allowed for mendelian randomization studies to provide insight into interactions between drug effects [6], as well as potential mediating mechanisms [7]. Given the tremendous advantages offered by genetic interrogation of drug target effects prior to clinical exploration [8], applications of mendelian randomization in this space are certain to continue their expansion.
